# Genome Sequence of *Saccharopolyspora erythraea* D, a Hyperproducer of Erythromycin

**DOI:** 10.1128/genomeA.00718-13

**Published:** 2013-09-19

**Authors:** Wei-Bing Liu, Wen-Bang Yu, Shu-Hong Gao, Bang-Ce Ye

**Affiliations:** Laboratory of Biosystems and Microanalysis, State Key Laboratory of Bioreactor Engineering, East China University of Science and Technology, Shanghai, China

## Abstract

*Saccharopolyspora erythraea* is a Gram-positive bacterium that can produce antibiotics. However, this microorganism must often be genetically improved for higher production before it can be used in an industrial setting. Here, we report the whole-genome sequence of the industrial hyperproducer strong mutator *Saccharopolyspora erythraea* strain D.

## GENOME ANNOUNCEMENT

*Saccharopolyspora erythraea* is a soil dwelling, Gram-positive, GC-rich bacterium originally identified as *Streptomyces erythraeus*, but it was later assigned to the genus *Saccharopolyspora* (1). *S. erythraea*, as an excellent producer, produces a large number of secondary metabolites with biological activities, including some used as antibiotics. However, this microorganism must often be genetically improved for higher production before it can be used in an industrial setting. Historically, strain improvement has been empirically carried out by multiple rounds of random mutagenesis and screening (2). More recently, these rational strain improvement strategies benefit from the support of genomic, transcriptomic, proteomic, and metabolomic technologies (3–6). Since 2007, when the complete genome sequence of *S. erythraea* strain NRRL 23338 was completed (7), many researchers analyzed the difference between NRRL 23338 and mutants at the genome and transcriptome level using microarray and comparative genomics technologies, as well as other methods (8–12). The whole-genome sequencing of *S. erythraea* will help scientists to better understand the regulation of the erythromycin biosynthesis pathway and its relationship to other genes, which will in turn allow for more sophisticated strategies for improving the production of erythromycin. According to the Genomes OnLine Database (GOLD) (13), genome sequencing of the industrial hyperproducer strain of this organism has not been done yet, and there is only one completed and one draft sequence available for *S. erythraea*, specifically, that of the model strain *S. erythraea* NRRL 23338. Here, we report the whole-genome sequence of the industrial hyperproducer strong mutator *S. erythraea* D. The analysis of the sequence may help us to gain further insight into the overall genetic variation in *S. erythraea* and to understand the mechanism of its high production of erythromycin in order to investigate strategies for further improving the strain.

Genome sequencing was performed using the Roche GS Junior technology. Roche 454-GS Junior generated 167,320 reads (average length, ~519 nucleotides) that provided ~10× coverage of the entire genome. Several assembly procedures were applied, and manual editing was performed with the 454-GS Junior data. The genome was annotated using the IGS Annotation Engine (14), and the NCBI Prokaryotic Genomes Annotation Pipeline (PGAP) 2.0 was employed for submission (http://www.ncbi.nlm.nih.gov/genome/annotation_prok/).

The final assembly contains 384 contigs, with an N_50_ contig length of 37,346 bp; the largest contigs assembled measure 152,774 bp. The genome of *S. erythraea* D is composed of 79,374,806 bp, with an average G+C content of 71.23%. Compared with NRRL 23338, 304 high-confidence mutations were found, in which 100 nonsense mutations are located in a coding region, 141 sense mutations are located in a coding region, and there is one small fragment insertion/deletion, 59 mutations, and three small fragment insertions/deletions located in a noncoding region.

A more-detailed comparative analysis of this genome with those of other mutant and NRRL 23338 strains will provide further insight into the specific properties of this high producer of erythromycin.

### Nucleotide sequence accession numbers.

This whole-genome shotgun project has been deposited in DDBJ/EMBL/GenBank under the accession no. AVCN00000000. The version described in this paper is the first version, AVCN01000000.
